# Experimental Approaches for Testing the Hypothesis of the Emergence of Life at Submarine Alkaline Vents

**DOI:** 10.3390/life11080777

**Published:** 2021-07-31

**Authors:** Thiago Altair, Luiz G. F. Borges, Douglas Galante, Hamilton Varela

**Affiliations:** 1 São Carlos Institute of Chemistry, University of São Paulo, São Carlos 13560-970, Brazil; 2 Brazilian Synchrotron Light Laboratory (LNLS), Brazilian Center for Research in Energy and Materials (CNPEM), Campinas 13083-100, Brazil; luiz.borges.pf@gmail.com (L.G.F.B.); douglas.galante@lnls.br (D.G.)

**Keywords:** origin of life, hydrothermal vents, bioenergetics, catalysis, hydrothermal reactor, electrochemistry, interface

## Abstract

Since the pioneering experimental work performed by Urey and Miller around 70 years ago, several experimental works have been developed for approaching the question of the origin of life based on very few well-constructed hypotheses. In recent years, attention has been drawn to the so-called alkaline hydrothermal vents model (AHV model) for the emergence of life. Since the first works, perspectives from complexity sciences, bioenergetics and thermodynamics have been incorporated into the model. Consequently, a high number of experimental works from the model using several tools have been developed. In this review, we present the key concepts that provide a background for the AHV model and then analyze the experimental approaches that were motivated by it. Experimental tools based on hydrothermal reactors, microfluidics and chemical gardens were used for simulating the environments of early AHVs on the Hadean Earth (~4.0 Ga). In addition, it is noteworthy that several works used techniques from electrochemistry to investigate phenomena in the vent–ocean interface for early AHVs. Their results provided important parameters and details that are used for the evaluation of the plausibility of the AHV model, and for the enhancement of it.

## 1. Introduction

Since the first half of the twentieth century, questions on the origin of life have changed towards strongly scientific and experimental approaches, which replaced logical speculations [1,2]. This has resulted in a considerable number of hypotheses [3,4,5,6,7,8] and some of them have proved to be developed enough to be tested by experimental approaches. The first known scientific and repeatable experimental protocol to test a hypothesis for abiogenesis is the remarkable Urey–Miller setup [1,2,9], which established the beginning of the so-called prebiotic chemistry. Therefore, in this review, we focus on describing and analyzing the experimental works designed to test an important hypothesis for the origin of life that was developed by the end of the 1980s: the alkaline hydrothermal vent model (AHV model), or submarine vent theory. This model is noteworthy because of its multidisciplinary conceptual framework and the resulting variety of experimental approaches that are based on it.

The current overall picture of the research in the field is marked not only by experimental approaches but also by the fact that the concept of origin of life (OoL) became less primary due to the unifying concept of emergence of life (EoL) [2]. In this context, the critical role of non-equilibrium thermodynamics and the proposal of a seed condition for the continuous evolution of life is recognized, and has guided not only the hypotheses but also the experimental works. The AHV model for the EoL attempts to give high importance to concepts related to non-equilibrium thermodynamics, highlighting continuity as an important property of the phenomenon of emergence. Thus, the model supports the mechanism of chemiosmosis—universally used in cellular systems—as key for life since its first steps in the transition from non-living matter, while recommending experiments to test the possible components for the emergence of that mechanism [10].

Alkaline hydrothermal vents (AHVs) are geologic arrangements that result from serpentinization, a chemical phenomenon that occurs on the seafloor [11,12], which basically consists of an oxidation reaction of the mineral content of the seafloor, such as olivine, exposed to water due to deep crustal faults [12,13,14]. Examples of these types of arrangements were discovered in 2001. They became known as Lost City hydrothermal fields (LCHF) [15,16]. Other hydrothermal fields of the same kind are now known [17]. The LCHF was discovered during an expedition aboard the ship Atlantis [15]. The discovery showed a huge local biological diversity [15,18]. These moderate-conditioned hot springs were already deduced as a likely place for the stability of organic compounds necessary to the EoL [19]. Hence, they are estimated to have existed in larger numbers on the early Earth, especially when compared to other submarine vents with more severe conditions, such as black smokers [20].

The AHV model arose in a debate context in which two main approaches that aimed to unravel the first steps of life on Earth prevailed. On the one hand, the remarkable proposal based on the primordial soup theory, or primordial organic soup model, was still very influential. This proposal, alluding to the famous 1871 letter from Darwin to Hooker [1,21], is commonly associated with the works by Oparin (1924) [22] and Haldane (1929) [23] and describes the production of organics in prebiotic Earth conditions and their accumulation in early ocean water. On the other hand, there was the more recently developed “surface metabolism theory”, proposed by Wächtershäuser [24,25,26,27,28], that provided another perspective for the problem. The latter has contributed to important discussions for the AHV model, as we will show later. The importance of the surface metabolism theory can be appreciated mainly by considering its emphasis on iron–sulfur minerals as a promising precursor of a CO_2_ reduction enzyme [19,29,30].

The AHV model originally aimed to focus on geochemical and geological details of the initial steps of the EoL, an approach that was not receiving appropriate attention at the time [30]. However, since then, the application of concepts related to complex systems and interface sciences was incorporated in the model. Consequently, a more complex and complete picture of the transition from non-living to living matter on the early Earth was being developed. In addition, this model provides a testable background for different areas of experimental works. Since then, several experimental works were conducted to tackle important questions based on the AHV model. For instance, several elements of the hypothesis began being tested, such as the geochemical background for the AHV environment, the catalytic properties of minerals proposed to have been present in early vents and the phenomena in the modeled vent–ocean interface, such as gradient formations and their coupling as drivers of prebiotic reactions. 

Therefore, the main objective of this review, as mentioned above, is to provide an overall perspective of the experimental approaches based on the AHV model for the EoL on the early Earth. The main general objective is to show the importance of experimentation for testing and fine-tuning the model. Consequently, we highlight the importance of reviewing the results. We also provide directions for further experiments; mainly those using the AHV model, which has a high number of publications proposing conceptual models and reviewing them. Hence, there is still a lack of reviews on the several experimental protocols that have already been developed based on tests of the model. Finally, in order to provide a background for the ongoing discussions, we will briefly present the context of the debate of the OoL studies in which the AHV model was developed. Thus, we present here the main conceptual elements of the model, primarily those that were commonly applied to the experimental works that have been developed so far. Lastly, we will detail the described experimental approaches, their setup and key results, as well as the trends for future works.

### 1.1. Context of Origins of Life Studies and Bases of the AHV Model

The question of the origin of life can be considered a relatively recent topic in the history of science. Since the well-known works from Miller–Urey [9], a testable experimental protocol has been created; a kind of approach that requires a respectable conceptual background [1,2]. These works, which resulted in remarkable experimental setup and results, were developed considering the theoretical background developed in works by Alexander Oparin [9,22]. In his most famous work, Oparin developed the theory of the transition from non-living to living matter due to successive events of synthesis and the accumulation of organic compounds in a prebiotic scenario. Moreover, another proposal for organic synthesis and accumulation on the early Earth was proposed, independently, by Haldane (1929) [23]. In the latter, UV radiation was emphasized as a trigger for the atmospheric CO_2_ reduction into organics that would accumulate in the early ocean, resulting in a so-called hot diluted soup. Because of this analogy, both hypotheses are commonly compared and known as the prebiotic soup theory. Both proposals also mention the necessity of a boundary in one of the intermediary steps in the transition process of non-living to living matter. Otherwise, the organics formed would simply diffuse throughout the early ocean. Usually, this boundary would be formed by aggregates known as coacervates [22,23].

The Miller–Urey experiment was considered to be, as mentioned, a landmark in the experimental approaches performed in the context of the OoL studies. It was also considered to be the establishment of prebiotic chemistry as a field of research [1,31]. In this experiment, the early ocean–atmosphere system was simulated based on the conditions proposed by Urey (1952) [32]. Thus, it comprised an apparatus in which a highly reducing atmosphere under 1.5 bar—consisting of CH_4_, NH_3_, H_2_ and water vapor—was submitted to electric sparks under the potential of 6 × 10^4^ V for a week [1]. As a result, organic compounds, amino acids among them, were detected in the solution [9,31,33]. Revisions were made in the conditions of the experiment due to new discoveries about prebiotic Earth conditions, mainly in the Hadean atmosphere. As a result, amino acid formation was still detected in several models of the early atmosphere [4,34].

To date, there is a consensus on the idea that to answer the question of the origin of life as we know it, a proposal must include the problem of organic matter formation [35,36]. Besides, the region at which life could have emerged is another important issue, as it will determine the sources of energy that played a role in this event, plus the presence of water and organic matter [3,4,6,37,38]. Thus, submarine hydrothermal systems compose a type of environment that was considered promising for the OoL [3,6,39,40]. These systems, also known as “hot springs” in the literature, were discovered in 1979, and very severe conditions were detected on the first ones that were reported [13,16,40,41]. They showed a very acidic (pH~2) and hot submarine environment (T~350 °C) due to their proximity to magma chambers, which also provided constant geothermal energy. Moreover, extremophile life forms were present [42,43]. 

Considering the Ool hypotheses on hydrothermal systems, an important one was proposed by Günter Wächtershäuser. It became known as the “surface metabolism theory” [26,28] also known as “iron–sulfur world” [2,4,25,44]. This hypothesis shares some aspects with the AHV model, mainly when considering the first publications on the latter. The similarities lie not only in the importance attributed to iron minerals surfaces—which are primary for both hypotheses—but also in the shared important insights that try to present a logic for the EoL on the early Earth.

The surface metabolism theory assumes the perspective that chemolithoautotrophy must be the key to our understanding of the first metabolism. It is based on a top-down approach that suggests that ancient levels of the phylogenetic tree are extensively composed of chemoautotrophs and thermophilic (considering that the first main references on the theory of surface metabolism mention examples of the prokaryotes whose optimal growth occurs at temperatures above 80 °C, it is more adequate to use the term “hyperthermophilic”, according to the *Encyclopedia of Astrobiology* (2015) [45]) organisms [27]. In addition, it considers the acetyl-CoA synthesis pathway as the most ancestral carbon fixation route for the known living organisms. This pathway, as detailed in the next section, depends on key enzymes that have iron–sulfur minerals as active centers [35]. In conclusion, it proposes a metabolism-first EoL. It would be based on a redox process involving a CO_2_-reducing reaction on the surface of an iron–sulfur mineral, commonly mackinawite (FeS), forming pyrite (FeS_2_) in a low pH hydrothermal condition (Equation (1)). Because of the chemisorption of organic molecules formed on the positively charged surface of pyrite, the system would be spatially bound in two dimensions, which would make the increase of molecular complexity possible. This could cause a protometabolic reaction network followed by a local increase of complexity [28]. An experimental approach based on this surface metabolism theory has also been developed [35,46,47,48,49].

(1) $$\mathrm{FeS}+H_{2}S\to {\mathrm{FeS}}_{2}+H_{2}$$

The sulfur metabolism and other hypotheses related to acidic and hot hydrothermal systems have some elements in common with the AHV model. However, some works have already shown that organics that are essential for life would not be stable in severe conditions, such as those known for hydrothermal vents [19,50]. Thereby, harmless, mildly conditioned hydrothermal systems, far from magmatic chambers and from plate tectonics boundaries (off-ridge vents), were then considered for the model. These types of systems have been anticipated based on the known serpentinization reaction [20,30]. Besides, AHV environments were estimated to be more frequent on the early Earth compared to the modern day [20,30,51]. Nevertheless, the AHV model was initiated as a primarily geochemically detailed model based on these alkaline, H_2_-rich and mildly warm hydrothermal systems. After the first works, elements from areas other than geochemistry were assimilated. Finally, the main milestone that supports the feasibility of the model is the discovery of the first submarine alkaline hydrothermal system, similar to the anticipated mentioned system, the LCHF.

## 2. The Conceptual Background for the EoL in Alkaline Hydrothermal Systems

The group formed by Michael Russell, Allan Hall, and collaborators, based on what was known about the serpentinization reaction of ultramafic rocks in the seabed [19,52], anticipated the AHV systems and proposed them as optimal sites for the EoL. Then, the discovery of the LCHF was crucial in order to consolidate the feasibility of the theory that was being developed. Since then, the AHV model has stood out in the literature [5]. Since the first articles, the fundamental question of the EoL has been the one concerning the functionality of living systems, rather than their defining nature [30].

Generally, we may separate the works related to the AHV model into three major categories. They are used here simply as means to discuss the experimental setup that is based on the model. They are not intended to be definitive categories, nor are they endorsed by the primary groups that are currently working on this topic. Until the 1990s, the papers focused on providing a geochemical background for the development of the model. Additionally, they described the now well-known grape-like (botryoidal) interconnected chambers scenario that results from alkaline hydrothermal fluid inflation. Since the late 1990s, and mainly after the discovery of the LCHF, a noteworthy aspect in scientific publications has been the emphasis on bioenergetics, based on the chemiosmotic coupling in the interface between the hydrothermal environment and the early ocean. In addition, these works have inspired most of the experimental works that followed, as well as variants of the model and parallel hypotheses, such as the geoelectrochemical and the chemiosmotic models for the EoL. However, recent works have shown trends in emphasizing the importance of considering living organisms as engines, consequently focusing on the mechanisms of entropy conversion. Therefore, in this last period, proto-molecular machine mechanisms were proposed, focusing on the details of the concept of a free-energy converter. Furthermore, these more recent works focused mainly on the presence of green rust minerals at the vent–ocean interface, in contrast with the ocus on iron sulfides that characterized the previous period. Green rust minerals have complex structures and behavior, which results in peculiar interfacial properties. Thus, it is supported that these properties mimic cellular transmembrane transport molecular machines.

### 2.1. Overall Geochemical Context in the AHV Model

As a background for the experimental works reviewed here, some basic aspects of the geochemistry of submarine AHVs and the ocean are discussed, especially regarding models for these scenarios on the Hadean Earth. Therefore, this section discusses the conditions that are commonly mentioned in the articles that will be detailed later. These include the basic chemistry that results from the serpentinization reaction, the composition of the surface of the vents mounds and the basic composition and physical chemistry of the deep early ocean.

As briefly mentioned, the AHV is a type of a hydrothermal system that resulted from the products of the serpentinization reaction. The simplified chemical representations for the reaction usually show the oxidation of olivine, a group of magnesium–iron silicate minerals, usually forming serpentine (a hydroxylated magnesium–iron silicate mineral), magnetite (Fe_3_O_4_) and molecular hydrogen (H_2_). However, some representations also include the formation of brucite (Mg(OH)_2_) and methane, or other small organics, as shown in Equations (2) and (3) [39,53]. As a consequence of the exothermal serpentinization reaction, a local alkaline (pH ~ 10–11) and warm condition (T ~ 70–150 °C) is generated in the form of hydrothermal fluid. The contact of the hydrothermal fluid with the ocean, which contains diluted CO_2_, results in the spontaneous formation of carbonate mounds [30,54]. This process is a natural analog of the injection method for chemical garden formation (see Section 3.2.1). Due to the local composition of the modern deep ocean, these mounds—composed mainly of calcite, brucite, iron brucite and aragonite—have a porous surface and a gradient-rich interface with the ocean [12,16]. However, as will be discussed later, it is expected that early AHVs had mounds richer in iron minerals, and also had brucite and Mg-clays [20].

(2) $$6{\mathrm{Mg}}_{1.5}{\mathrm{Fe}}_{0.5}{\mathrm{SiO}}_{4}+7H_{2}O\to 3{\mathrm{Mg}}_{3}{\mathrm{Si}}_{2}O_{5}{\left(\mathrm{OH}\right)}_{4}+{\mathrm{Fe}}_{3}O_{4}+H_{2}$$

(3) $${\left(\mathrm{Mg},\mathrm{Fe}\right)}_{2}{\mathrm{SiO}}_{4}+H_{2}O\to {\mathrm{Mg}}_{3}{\mathrm{SiO}}_{5}{\left(\mathrm{OH}\right)}_{4}+\mathrm{Mg}{\left(\mathrm{OH}\right)}_{2}+{\mathrm{Fe}}_{3}O_{4}+H_{2}+{\mathrm{CH}}_{4}+C_{2}-C_{5}$$

The precise composition of the early ocean from ca. 4.4 to 4.0 Ga, which is often mentioned in works describing the AHV model [30,55,56,57], is still debatable. However, the qualitative description and the constraints of the early ocean chemistry—which form the bases of the experimental works that seek to simulate the vent–ocean environment—are more strongly supported. Because of its high carbonic concentration, the early ocean is considered mildly acidic (pH ~ 5.5) [57,58]. Hence, a moderate to cold local temperature in the deep ocean from 5 to 16 °C was postulated [19,59]; these are the assumptions that support the experimental works that are described here. However, some conceptual works mention higher local temperatures, up to 90 °C [30] next to the vents. In addition, the presence of Fe, Mo, Zn, W, Co and Ni (concentrations: Fe >> Zn > Ni ~ Co ~ W) in concentrations higher than those found in modern oceans, as an effect of the presence of acidic springs, is often emphasized [30,56]. Finally, for the AHV model, other aspects in the Hadean scenario are considered, in agreement with several other groups, mentioning an anoxic atmosphere, high UV flux on the surface and higher hydrothermal and volcanic activities [60,61].

### 2.2. Concepts from Complexity Sciences and Far-From-Equilibrium Thermodynamics in the AHV Model

From the perspective of the AHV model, it would be unsustainable that, in its origin, life would use material resources and mechanisms for energy drastically different from those of modern systems. This model contrasts with classical approaches, which emphasize the presence of energy sources—while considering that they are intense and frequent in a given environment—and the formation and aggregation of the so-called building blocks [62]. Several hypotheses state, for example, that these earlier proposals present the requisites for life’s origin based on mechanisms such as those that rely on high voltage electrical discharge, geothermal heat, wet-dry cycles or UV radiation. Thus, the main issue for life emergence in the AHV model seems to be the emergence of “engines”, which is based mainly on the work by Cottrell (1979) [63]. He used as background the concepts of the far-from-equilibrium thermodynamics’ school developed by Prigogine and the synthetic biology from Leduc (1911) [64]. The details of these concepts of engines applied for the EoL are presented, mainly, in two important references by Elbert Branscomb, Michael Russell and colleagues: “Turnstiles and bifurcators: The disequilibrium converting engines that put metabolism on the road” (2013) [56] and “Escapement mechanisms and the conversion of disequilibria; the engines of creation” (2017) [5]. 

There is widespread agreement on the difficulty of a precise description of the nature of living systems. This means a lack of a clear boundary between what can be defined as a living system among the non-living ones [3,4,65,66], which is a consequence of the immense diversity of structures and morphologies of the known living systems. Notwithstanding this issue, the aforementioned boundary has been considered gradual, continuous, and sensible since the general acceptance of the concept of Darwinian evolution [1]. In the work of Leduc (1911) [64], entitled “The mechanism of life”, this gradual, continuous and sensible notion for the living to non-living system transition is presented. In this pioneering work, Leduc seeks to describe the physical chemistry of living systems from their nature as open systems. He also suggests that the study of living systems is optimized if it is based on the properties of the interface between two water solutions, for this type of study is the core of the maintenance of cellular mechanisms [64]. This notion has turned into one of the basis and the key factor for the development of the AHV model of the EoL.

Self-organization is closely associated with emergence and is far from thermodynamic equilibrium and non-linear evolution [7,36,67,68,69,70,71,72]. This association is the reason the AHV model uses the concept of “emergence of life” instead of the famous “origin of life”. Consequently, for the emergence of a specific complex structure, a specific requirement is necessary. This means a framework of specific physical–chemical conditions and a preexisting structure, which is naturally expected to be formed by chance, so it could be a “seed” for continuous and dynamic evolution [56,57,73]. Therefore, according to the AHV model, the alkaline vents would be an optimal site that could fulfill the requirements for the EoL [62].

Besides the clear necessity of an environmental condition far from thermodynamic equilibrium for the maintenance of living systems, so they could be supplied with energy and matter, two other fundamental points are the entropy conversion and the gradient dissipation mechanisms [5,36,74]. The need that living systems have for far-from-thermodynamic-equilibrium conditions has already been proposed in classic works, such as Schrödinger’s famous book *What Is Life?* (1946) [70] and Boltzmann’s *Theoretical Physics and Philosophical Problems* (1974) [69]. Some concepts related to non-equilibrium thermodynamic systems are still under development, so the verification of diverse types of systems is still imperative. For instance, there are concepts, such as the maximum gradients dissipation and maximum entropy production, that still demand more detailed proofs for several types of systems [36,68,75,76]. From the perspective of the maximum gradients dissipation, living systems would help to more effectively dissipate the gradients in which the Earth system is embedded. Some authors argue that the high sophistication and complexity of these types of systems result in their effectiveness as gradient dissipators when compared to other non-living systems [59]. On the other hand, maximum entropy production is based on a numerical parameter, such as entropy production rate. This rate is proposed to reach a maximum value in a non-linear system when it prevails [75,76]. These principles are based on the second law of thermodynamics and are proposed as parameters of prevalence among multistable systems. They have also been proved for some classic systems. However, they have yet to be rigorously tested/experimented/discussed in the context of the EoL.

Beyond the struggle of entropy in living systems, as mentioned by Boltzmann (1974) [69], the AHV model emphasizes the notion of the necessary specificity of processes for the maintenance of life. For this reason, we also have to consider that the natural electrochemical gradient—which exists as a consequence of ion and redox gradients as driver forces, especially due to a difference of protons concentration—is considered as universal as the genetic code of living systems [77,78]. Considering all of this, electricity is once again associated with the origin of life. However, in this case, life would have originated in a specific form in chemiosmotic gradients, under low potentials (*ca.* hundreds of mV in average for transmembrane processes [30,79]), instead of from lightning or arc-lights with tens of thousands of volts. Finally, a study that used the AHV model has concluded that a useful electrochemical gradient was probably domesticated throughout the EoL process, and that key minerals were assimilated for the necessary coupling of the processes related to the maintenance of life [62].

Due to the driver force specificity, living systems require several specific far-from-equilibrium converters. The known modern living systems depend on highly complex molecular motors as converters, such as enzymes [5,80,81]. It is evident that this complex enzymatic system had simpler analogs that have evolved. Therefore, the AHV model supports the idea that the precursors of these complex molecular motors resulted from specific minerals, such as iron sulfides, sequestered by organic molecules [62]. Moreover, some studies have proposed the presence of minerals as important elements in the context of the EoL, either as reactive components [27,35] or due to their regular patterns [82]. In addition, the AHV model shares some important aspects with the surface metabolism theory (see Section 1.1)—e.g., the emphasis of the iron–sulfur minerals as active centers of prokaryotic cell enzymes [35], such as hydrogenase, ferredoxin, acetyl coenzyme-A synthase and carbon monoxide dehydrogenase [54]. Furthermore, another category of iron minerals has been mentioned in the most recent articles: the iron oxyhydroxide group, or green rusts [83], as will be described later in this work.

### 2.3. The Early Chemiosmotic Coupling and the Geoelectrochemical Driven EoL

As mentioned before, works based on the AHV model received a lot of attention after the discovery of the LCHF. Among them, there was an important contribution from those which focused on models of energy conversion mechanisms for the EoL in the sub-marine alkaline hydrothermal environment [39,51,84,85,86]. Moreover, they focus on the links between models for the chemistry of energy transfer in early AHV environments and the metabolism of microorganisms. The main link is based on the mechanism that is generally used by cells to harness energy involving ions gradients, according to the chemiosmotic theory. 

**Figure 1:**
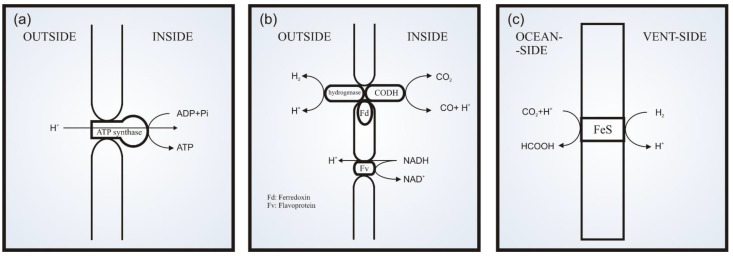
Comparison between the chemiosmotic mechanism on modern cells and the proposed mechanism for the vent-ocean interface: (**a**) typical setting for the chemiosmotic mechanism synthesizing ATP; (**b**) electron and proton transport coupled proposed for CO_2_ reduction on a membrane [92]; (**c**) proposed prebiotic chemiosmotic mechanism for CO_2_ reduction in a Hadean vent–ocean interface.

The chemiosmotic hypothesis was developed in the pioneering works by Mitchell. It would later gain the status of theory [87,88,89]. His works proposed a fundamental common mechanism in living cells coupling chemical and osmotic phenomena. They basically describe an anisotropic mechanism in which the pH gradient is mediated by amphiphilic membranes and coupled to molecular machines. Indeed, it was later discovered that this mechanism is as universal as the genetic code in living systems [77,78]. Thus, it is associated with energy transduction and harnessing in a number of processes that maintain living systems. Besides that, the chemiosmotic theory was originally based on the studies of what is now known as the adenosine triphosphate synthase (ATP synthase) system [87,88]. The membrane-located reversible ATP synthase produces ATP from adenosine diphosphate (ADP). It is driven by a proton electrochemical gradient, which is maintained by redox reactions in respiratory or photosynthetic electron transport that pumps protons across the mitochondria membrane (Figure 1a) [51,90,91].

Then, the chemiosmotic theory proposes a functional association of space-directed phenomena, coupling the chemicals to osmotic processes in metabolic pathways [88]. In this vectorial process [90] driven by natural electrochemical potentials, chemical groups are transported to different sides of a membrane, mediated by an anisotropic membrane-located catalytic carrier system (Figure 1a). Hence, the energy harnessed in the electrochemical gradient is transduced to metabolic processes or into chemical energy, which is the classical example in the process of the ATP synthase system [90]. In this case, common on membranes of mitochondria, bacteria and chloroplasts, the ATP synthase translocates protons induced by pH and/or electric potential gradients, while it phosphorylates ADP, forming ATP [88]. This occurs at the same rate as respiratory or photoredox chains, which are also proton translocating systems with the same polarity as the proton translocating of ATP hydrolysis back to ADP. In conclusion, a loop is created: the electrochemical potential resulting from the pH that is “consumed” in the ATP formation is regenerated in a redox chain metabolism.

Finally, an important variant of the AHV theory is strongly based on concepts from Mitchell’s chemiosmotic theory. In this variant, besides the chemical richness and the non-equilibrium condition of the AHV environment, it is its special setting that makes it promising for crucial steps of the EoL. In short, that environment holds similarities with the chemiosmotic bioenergetic core of organisms [51]. Due to the precipitation of minerals that form the mounds, AHV environments keep pH, redox and thermal gradients mediated by inorganic membranes/barriers (Figure 1c) [29,58,93]. Thus, these gradients create a natural electrochemical potential that would drive the endergonic processes among components in the hydrothermal and oceanic environments. However, unlike the setting in modern cells, neither a proton nor any kind of ion pump for keeping the gradients would be necessary, since non-equilibrium is maintained by the serpentinization-produced chemical condition [51]. Moreover, and most importantly, some works support the hypothesis that, in early AHVs, those inorganic membranes shared vital properties comparable to membranes of known cells. These membranes are related to the anisotropic enzymatic molecular machinery that is attached to modern membranes. 

Several models estimate that the early ocean had a much higher salinity than today’s oceans. Besides, it is estimated to have contained much more Fe^2+^ ions, because of the higher solubility of this ion and due to the activity of black smokers [20,30,94,95]. This hypothesized early ocean condition allows for the stabilization of mixed-valence iron–sulfur or oxyhydroxide minerals. Iron–sulfur shows its importance due to its prevalence in active centers of metalloenzymes [54,96]. As a special example, ferredoxin catalytic proteins present in carbon monoxide dehydrogenases are known for their iron–sulfur electron relay centers [97,98]. Besides, these bifunctional enzymes contain Ni and function as reaction centers of acetyl-CoA synthase and carbon monoxide dehydrogenase (ACS/CODH) [93,99,100,101]. Moreover, iron oxyhydroxide minerals, or green rusts, have a similar structural configuration when compared to hydroxy bridging present in di-iron methane monooxygenase enzymes [83,98,102]. 

The acetyl-CoA synthesis pathway (Wood–Ljungdahl pathway, WL) is an important reference for the proposal of the emergence of bioenergetics and carbon fixation metabolism. Its modern biochemistry involves reaction steps catalyzed by iron [—nickel] sulfide centers in enzymes [26]. Considering that, this pathway is inferred as the most ancient and simple for carbon fixation and energy metabolism without ATP [26,85,103]. Thus, in a proposed protometabolic pathway in early AHVs, CO_2_ in the early ocean is reduced by a serpentinization-produced electron donor, such as H_2_ or CH_4_ [51]. Furthermore, the reduction is catalyzed by iron–sulfur minerals, such as greigite, mackinawite and its Ni-bearing variants [98,104]. In this context, the pH and redox gradients are, similarly to those in the modern WL processes, the driver forces of the mechanism [78]. Besides the WL analog, the thermal gradient in the AHV setting is proposed to help the fluid dynamics that would keep the AHV as a reactor. Thus, an analogous protometabolic pathway is proposed. Its first step would be the same pathway of WL, which limits it. It consists of CO_2_ being reduced by H_2_ into formic acid on an iron–sulfur surface under an electrochemical gradient as the primary driving force.

## 3. Experimental Setups and Results Investigating the AHV Model

Besides the development of its conceptual framework, the AHV model has also been experimentally tested since the beginning. In fact, this experimental approach is currently a very active research area. On the one hand, there is the group of experiments that explored the geochemical and chemical perspectives of the AHV scenario. On the other hand, there is the prevalence of experiments that explored the interface phenomena. The latter use techniques and concepts from electrochemistry and material sciences. As usual in OoL studies, most of the experimental works reported here seek to simulate the condition and the physical setting proposed in the hypothesis being tested. However, as will be described in detail, several experimental studies were performed to enlighten specific phenomena or parameters. The protocols concerned with the geochemical and chemical parameters, as well as their reactors and benchmarks, are usually developed from zero. In contrast, in the application of a known setup, adapting specific parameters is usual for the experiments exploring interface phenomena.

### 3.1. Reactors Simulating AHV Chemistry and Geochemistry

#### 3.1.1. Hydrothermal High Pressure Flow Reactors

**Figure 2:**
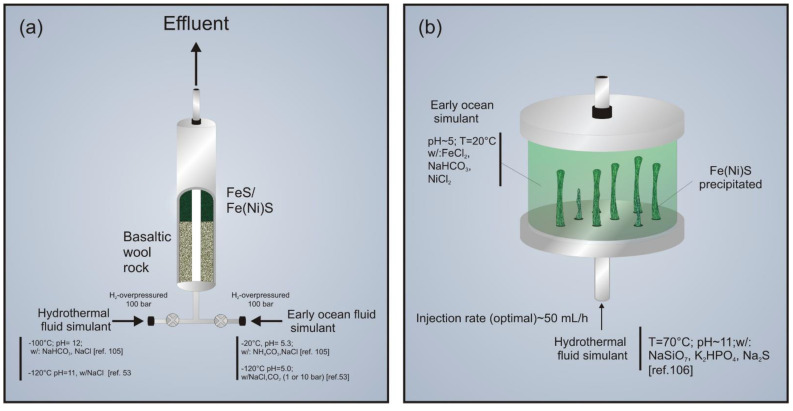
Visual illustration of the chemical reactors simulating early vent-ocean environment: (**a**) the reactor column from the hydrothermal setup developed by Mielke et al., 2010 [105] and adapted by White et al., 2020 [53] for simulating the contact of hydrothermal fluid, early ocean fluid and the Hadean crust under high pressure; (**b**) the origin-of-life reactor, as proposed by Herschy et al. (2014) [106] main vessel for testing the formation of Fe(Ni)S barrier and its properties for organic formation.

Mielke et al. (2010) [105] developed a reactor to simulate the chemical water–rock interactions mimicking a natural system. In it, the hydrothermal solution flows through veins and pods beneath the analog of the ancient ocean floor and then mixes with the analog of early ocean water when it rises from the floor (Figure 2a). However, the setup can be adapted for simulating hydrothermal interactions in another wet and rocky planet. The setup consists of two stainless steel cylinders used as solution reservoirs, both capable of reaching temperatures up to 140 °C and high levels of pressure (>100 bar). The first cylindrical vessel is filled with the H_2_-rich, pH 12, hydrothermal fluid, and the other with the pH 5.3 Hadean ocean solution, both of them in anoxic condition. The vessels then lead to the reactor, which is fed by the fluids driven by hydrogen overpressure. In the reactor, a quick conversion of the acidic early ocean solution into a highly alkaline solution was detected. Its pH oscillated between 11 and 12.3 and was caused by the dissolution of basalt rock wool simulating the Hadean ocean crust [105]. An analysis of effluent showed the formation of hydrogen sulfide ions (${\mathrm{HS}}^{-}$), and an oscillation of sulfide concentration around 25–50 ppm after 100 min of the interaction in the reactor. In addition, the concentration of calcium ions dropped, which led to calcite precipitation. The iron concentration was kept constantly below the detection limit of 0.1 ppm. Furthermore, analysis of the rock wool alteration due to these reactions showed a loss of calcium and aluminum, as well as an overgrowth of iron sulfide layers and green rust (iron oxyhydroxide). Finally, the effluent from this hydrothermal reactor was used to simulate hydrothermal mixing with ocean waters. As a result, precipitates known as chemical gardens were formed (see Section 3.2.1). One of the many advantages that this system provides is the capability of inducing a mix of liquid and gas phases into the reactor without pressure loss. However, it does not allow the control of the flow rate in the withdrawal of both vessels, which implies an unknown proportion of fluids mixture, even with rigorous pressure control.

Using an adapted version of the reactor developed by Mielke et al. (2010) [105], White et al. (2020) [53] investigated the formation of organic compounds during the serpentinization process (Figure 2a). In the adaption, ground pentlandite and komatiite were placed together with the basaltic wool as the simulation of the Hadean ocean crust. Moreover, there was the addition of CO_2_ under different pressures (from 0 to 120 bar) to the Hadean ocean simulant. Each fluid was passed through the reactor for 4 h. Pentlandite was used as an iron sulfide predicted for early AHVs, and has shown catalytic properties for CO_2_ reduction, as described in Section 3.2.2. In the effluent solution, concentrations of Ca, Mg, Si and ${\mathrm{HS}}^{-}$ were detected, along with a final high pH, similar to the results from the original reactor setup. However, in this adapted setup, organics such as formate (${\mathrm{HCOO}}^{-}$), acetate ($H_{3}{\mathrm{CCOO}}^{-}$) and methane (${\mathrm{CH}}_{4}$) were also detected in the effluent, though in minor quantities. In addition, the experiment detected hematite, magnetite and iron sulfides, such as mackinawite (FeS), pyrrhotite (Fe_7_S_8_) and pyrite (FeS_2_). They are found in the reactor after the alteration of the sulfide-bearing rocks. 

#### 3.1.2. The Origin-Of-Life Reactor

Herschy et al. (2014) [106] developed an open-flow reactor to test the hypothesis of the formation of an iron [–nickel]–sulfur barrier in the interaction between alkaline vent fluids and the early ocean (Figure 2b). Moreover, this setup was used to test the hypothesis of the reduction of early oceanic CO_2_, catalyzed by the iron–nickel sulfide under hydrothermal conditions [54,106]. The simulated alkaline hydrothermal fluid (pH ~ 11, 70 °C) was injected into the reactor vessel in a mildly acidic early ocean simulant (pH ~ 5, 20 °C). The mixture of both fluids, at room pressure, led to the precipitation of Ni-bearing mackinawite. In addition, the formation of organics, expected as catalyzed CO_2_ reduction products, was detected. However, the above-mentioned work aimed to provide preliminary results that would open new pathways for further works to enhance experimental protocols. Thus, some type of optimization for monitoring CO_2_ reduction, such as carbon labeling, was assumed to be necessary. 

To precipitate a semi-conducting Fe(Ni)S barrier, alkaline fluid was injected into the vessel in the presence of an acidic environment (pH ~ 5, 10 °C) in order to simulate the acidic ocean. The mixture of both fluids led to the precipitation of a vertical tube structure composed of ferrous silicates, phosphates, and a small amount of Fe(Ni)S. The authors assumed that these thin-walled structures were needed to perform the role of a semi-conducting barrier in order to reduce CO_2_, leading to the increase of the natural proton gradient. The experiment has shown the formation of several organic compounds, such as 1-propanol, formate and formaldehyde. The work provided empirical evidence that hydrogen combined with carbon dioxide using Fe(Ni)S as a catalyst can form organics using a natural proton gradient under mild conditions of temperature and pressure.

### 3.2. Interfacial Chemistry and Electrochemistry as Key Factors for the Emergence of Life

As highlighted in Section 2, the AHV model has emphasized, since its beginning, the importance of interface phenomena as a key factor for electing the AHVs as promising sites for seeding the emergence of life. Considering the omnipresence of chemiosmotic coupling for life as we know it, and given the theoretical bases of biophysics and its emphasis on engines for bioenergetics, experiments to test the hypothesis of the EoL related to these perspectives have become even more important recently. Thus, these experiments seek to simulate aspects in the interface between two solutions in a vent—ocean interface on the early Earth. 

The experimental works described explore elements of this interface, such as the motive force of the gradients, the mechanism of the precipitation of the hydrothermal mound and the electrochemically activated mineral surface present in early AHVs. In addition, the detection of electrical currents in natural deep-sea hydrothermal systems [107,108] was the basis for the development of the geoelectrochemical-driven OoL model [99]. This model—influenced by both the AHV model and the surface metabolism hypothesis—puts a strong emphasis on the interfacial phenomena and mainly on the electrochemistry of minerals under hydrothermal conditions.

#### 3.2.1. Chemical Gardens and the Simulation of Early AHVs

Chemical gardens are self-organized chemical systems that result from a combination of buoyancy, osmosis and chemical precipitation [109,110]. Their morphology has been associated with biological systems, biomimetics or a plant-like structure since they were first reported in the literature [109,110]. Furthermore, in the context of the OoL studies, chemical gardens were considered by Leduc and Herrera as important systems due to their morphology [64,111]. The forming conditions of these self-assembled structures are already known, as well as several of their properties, given the several examples of these kinds of systems. Traditionally, most of the research involving chemical gardens was based on morphological studies of the precipitated structures [112]. Nevertheless, the investigations of chemical gardens in works linked to the AHV theory are included in the works that seek to explore their other important properties. The composition, membrane potential and catalytic properties of iron sulfide and iron oxyhydroxide chemical gardens are explored under conditions analogous to submarine alkaline vents on the early Earth.

**Figure 3:**
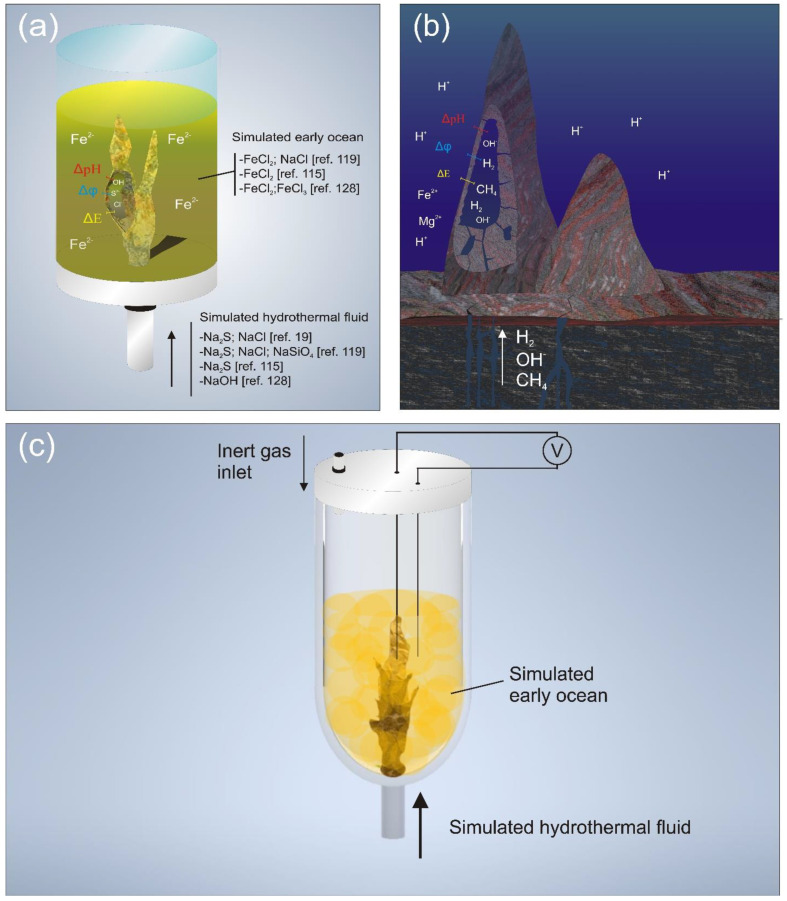
The analogy between iron–sulfur chemical gardens in a laboratory experiment (**a**) and an AHV mound on the Hadean ocean floor (**b**) regarding the generated natural membrane potential (∆E) as a consequence of pH gradient (∆pH) and redox gradient (∆ϕ). In addition, (**c**) a typically simplified scheme for the protocol that measures the membrane potential results in the values shown in Table 1.

Traditionally, chemical gardens are formed from a solid “seed” of crystal metal salts when dissolved in a solution containing different types of anions, such as aluminates, borates, carbonates or silicates [109,113]. The precipitation results in the chemical garden membrane surrounding the metal salt. Furthermore, there is an osmotic flow driven by the activity gradient generated across the membrane, followed by successive cycles of membrane rupturing, growth and precipitation. In another method, the membrane formation occurs due to the injection of a solution into another one, both being chemically contrasting. In this injection growth method, analogously to the seed growth, the precipitate membrane is formed at the interface between the two pH- and composition-contrasting solutions, directly out of the injection aperture (Figure 3a) [110]. The difference is that, in this method, osmosis is not the limit of the internal fluid pressure supply. This latter method is considered to offer a greater parameter control, and it is also the most commonly used in experiments that seek to investigate early AHVs. A process analogous to the injection method occurs in nature, which results in the formation of the structures in alkaline hydrothermal systems (Figure 3b) [19,30,114].

Chemical gardens may have several sizes, and alkaline hydrothermal vents are considered ten-meter-sized natural chemical gardens [115]. In their formation, the contact of hydrothermal fluid with the ocean water resulted in a process similar to the injection growth method for chemical gardens. Therefore, analogously to hydrothermal systems, chemical gardens are gradient-rich environments and explored as models for those systems in several works [79,112,116]. The precipitated membranes of chemical gardens are also analogously full of cavities, or even pores, showing several small compartments. This is an important property for the origin-of-life studies, mainly the AHV theory [10,117]. Moreover, across its precipitated inorganic membrane, pH and chemical gradients are kept and result in membrane potential due to the Gibbs–Donnan effect [110,115]. As it will be described in the following paragraphs, this potential in synthetic early AHV chimneys may assist surface prebiotic chemical reactions, or even generate electrical current, as a fuel cell-like behavior.

**The method for investigating early AHVs with chemical gardens**. The formation of chemical gardens from the injection method allows the reproduction of morphologies and mineralogies of hydrothermal systems [54]. In all works that have proposed experiments using this method, the injected solution is a model of the hydrothermal fluid, while the reservoir contains a model of the early ocean. Usually, iron mineral chemical gardens are formed for EoL studies, and the effects of the different chemistry and concentrations of the early ocean and hydrothermal fluids are demonstrated, just like the effects of different injection flow rates. Moreover, the investigation on their morphology and stability, pH and membrane potential are usually monitored as well. Membrane potential is an important parameter in most of the works that investigate several models for the early AHVs. In these experiments, electrodes were placed very close to each side of the precipitated membrane (Figure 3c) [116,118]. Besides, spectroscopy techniques based on x-ray and electron microscopy were used for investigating, respectively, the composition and details in morphology of the artificial chimneys [114,119]. In the following sections, details on works that considered chemical gardens as models to investigate properties of AHVs on the Hadean Earth will be discussed. 

**Formation of iron mineral chemical gardens and inorganic membranes under different conditions.** In the pioneering works, iron sulfide “chimneys” were formed and envisaged to be used in origin-of-life experiments. A pH >13 salty alkaline solution with NaCl and Na_2_S, injected in a pH 1.3 saltwater containing NaCl and FeCl_2_, resulted in a colloidal gel membrane forming globules and tubes [19]. The resulting iron monosulfide chimney showed properties comparable to those of hydrothermal orebodies [30,120]. The high concentration of Na_2_S (250 mmol L^−1^) has proved to be necessary for producing cavities, such as bubbles, in the precipitate [19,117]. In another approach, computer simulations indicated high supersaturation during the mixture of hydrothermal fluid in early ocean waters, which provides a background to the mechanism of membrane formation [55]. Moreover, it was shown that the relation between electrochemical potential and pH in the interface of both solutions explained the expected electrochemical potential that was generated. Once it was shown that the morphological and chemical analogies between hydrothermal systems and iron mineral chemical gardens were formed by injection growth, further studies explored the basic properties of these structures. As already mentioned, the variation of the chemistry of both the hydrothermal and ocean were explored to investigate the effects on the morphology and minerals present in the simulated mounds. Thus, chimneys were formed, injecting eluent of a reactor that simulates the water–rock interaction in early alkaline hydrothermal systems (see Section 3.1.1). The precipitated membrane showed a complex morphology that depends on the injection flow rate. It may vary from a predominance of fibrous holes to bulbous structures. These structures consisted of silicate, carbonate and iron-rich minerals, mostly green rust, and a minor part of iron sulfides. In addition, a more sulfide-rich environment was simulated, for it was expected in the Hadean Earth due to pyrrhotite-rich komatiitic rocks, estimated to both compose the deep ocean floor and even exist all over the planet’s surface [54,119,121,122]. Thus, the iron sulfide precipitated membrane was performed and tested under different chemical conditions and oxygen-free conditions. Besides, the sulfide- and silicate-rich alkaline solutions were used in temperatures between 25 °C and 70 °C—which is a typical average temperature of AHVs [12,15,20]—and injected into an iron (II)-rich solution.

Chimney-like structures with tens-of-µm-thick iron sulfide membranes were produced, and they were tested in several conditions of varying temperatures and pH. Under 25 °C, mackinawite (FeS) prevailed, along with iron (II) hydroxide, over the outer surface of the chemical garden produced. On the other hand, at higher temperatures, mackinawite oxidized, forming greigite (Fe_3_S_4_) [119]. The pH variation is also reflected in the FeS/Fe(OH)_2_ surface ratio, although causing a difference in the morphology and mechanical resistance of the chimney. In addition, the surface of iron sulfide chimneys showed a metastable condition, under which mackinawite turned into greigite at 70–75 °C [54], as shown in Equation (4). As metastable structures, mackinawite and greigite have proved to be interesting for catalysis, despite their tendency for oxidation [54].

(4) $$4\mathrm{FeS}+2H_{2}O\to {\mathrm{Fe}}_{3}S_{4}+\mathrm{Fe}{\left(\mathrm{OH}\right)}_{2}+H_{2}$$

**Prebiotic chemistry and the chemiosmotic theory for the EoL in chemical gardens and inorganic membranes**. Once procedures to form chemical gardens simulating early alkaline hydrothermal systems were established, the parameters and phenomena related to EoL studies started to be investigated. In this context, synthetic hydrothermal chimneys were tested as reactors in which prebiotic chemistry reactions and electrochemical potential could be investigated. An important assumption on which these studies were based is related to the properties of several mineral surfaces known to be catalysts of many reactions considered to be suitable candidates in the prebiotic scenario. 

The presence of peptides, plausible in the early ocean, has stabilized the iron mineral synthetic chimneys. They have also been shown to change the chimneys’ morphology and chemistry [119,123]. The structures, whose outer surface is mostly composed of mackinawite (FeS) and iron (II) hydroxide [Fe(OH)_2_], have shown an enhancement in their membrane durability, the development of crenulations on their outer wall and a change to their FeS/Fe(OH)_2_ proportion in the presence of pentameric peptides [119]. Changes in composition were also detected in iron sulfide-only chimneys due to the effect of the presence of small peptides or RNA [123].

Inorganic membranes were formed on iron sulfides/silicates, in a process analogous to those which form hydrothermal systems. Both present catalytic properties that aid in the formation of pyrophosphate. The iron sulfide/silicate inorganic membranes formed in processes analogous to those which form hydrothermal systems presented catalytic properties for the formation of pyrophosphate (PPi). This substance is [124], a universal energy currency in known living systems, and was proposed to be an ATP precursor [125,126,127]. The surface of the membrane concentrates orthophosphate (Pi), and catalyzes its condensation reaction, forming PPi, which is also concentrated on the surface. This condensation reaction only occurs in modern living systems mediated by the membrane-integrated H+-pyrophosphatase protein [124,125,126]. Besides, synthetic chimneys containing iron silicate sulfide also catalyze the oligomerization of adenosine monophosphate (AMP) ribonucleotide, resulting from dimers up to 4-mers [121]. Longer oligomers were detected using AMP activated by imidazole (IUPAC name: 1,3-Diazacyclopenta-2,4-diene) in the presence of uracil monophosphate (UMP). An overall improvement in oligomerization was detected by introducing montmorillonite clay [(Na,Ca)_0.33_(Al,Mg)_2_(Si_4_O_10_)(OH)_2_.nH_2_O] into the system. Finally, the catalysis of amino acid synthesis, such as alanine from pyruvate amination, was also detected on the surface of green rust synthetic chimneys [128]. 

For the chemiosmotic AHV model, the membrane electrochemical potential, along with other gradients, were important properties that were tested in synthetic chimneys, regardless of the surface composition and compartments in the precipitate. In publications about chemical gardens, even when lacking any EoL motivation, there are some examples of experimental works exploring the membrane potential. In silica gardens, a special arrangement for measuring the local pH and membrane potential was built. A potential from 20 to 120 mV and an increase of local pH were detected after several hours after the structure formation [112]. In addition, iron sulfide membranes have already been shown to keep pH gradients and generate electrochemical potential around them, due to chemical contrast in the forming fluids that are in contact [129]. Using growth injection for membrane formation, it was possible to detect the potential of iron phosphate silica chemical garden membranes, and a potential of up to around 150–250 mV was established [79]. In works designed to address the EoL problem, iron sulfide membrane potential was tested [115,116,118,129] for different chemistries for the fluid inside of the formed membrane. The membrane potentials obtained varied from 239 to 430 mV, and then they increased up to 923 mV when the fluid was substituted for a Na_2_S solution after the establishment of the membrane (Table 1) [118]. Different models of fluids and temperature resulted in the formation of different membrane compositions. Usually, the prevailing material was mackinawite, but they frequently contained greigite and green rust, too [115].

#### 3.2.2. Electrochemically Activated Mineral Surfaces

As discussed above, mineral surfaces are proposed as key players for the emergence of a chemiosmotic couple in the early AHV environment. Thus, this proposal has increased the number of experimental protocols that explore minerals that are often considered important for the AHV model, by the use of modern electrochemistry tools. Minerals investigated using these tools are mainly iron sulfides and Ni-bearing iron sulfides. Most of them were detected in experiments such as those reported in Section 3.1.1, and they are similar to active centers of metalloenzymes, as discussed in Section 2.2. Among them are minerals such as mackinawite (FeS) and its Ni-containing form, greigite (Fe_3_S_4_) and violarite (FeNi_2_S_4_). Some of these are considered key minerals [130] for the AHV and geoelectrochemistry models for the emergence of life. The CO_2_ reduction electrocatalysis of the minerals is the most common reaction that was investigated. However, ammonia formation from nitrate/nitrite was also explored. The carbon fixation from the CO_2_ reduction is supplemented with ammonia as a nitrogen source for nucleic acids, amino acids and other N-containing biomolecules [99].

Furthermore, some works had already explored some of the mentioned minerals in electrochemistry-related areas. Those that investigated the emergence of life found interesting properties for these minerals under several conditions. Hence, parameters such as potential, temperature and pH varied while products were detected, and it was found that proton-coupled electron transfer may occur [131]. These electrochemistry works drew conclusions about the properties and limitations of specific minerals, which provided perspectives for the enhancements of these models.

**Iron sulfide mineral electrochemistry**. The iron (II) monosulfide (FeSm), known as mackinawite, is a known metastable material and metallic conductor, and is common in aqueous environments [132]. The properties and environmental utility of this mineral are already known, even though they are not related to CO_2_ reduction [133,134]. Besides its importance—once already mentioned for the AHV model, which considers it a key mineral for the emergence of life [130]—there has been little exploration of its electrochemistry in experiments applying in the AHV model. Evidence has shown that mackinawite interacts with CO and CO_2_ under pH 6.8 conditions at room temperature [135]. However, the analysis was jeopardized because of the mineral sensibility at room conditions. 

On the surface of electrochemically synthesized mackinawite in a sulfide-rich solution, the effect of different potentials tested in a phosphate-buffered solution with pH 6.8, was observed [135]. Under this pH condition, mackinawite was estimated to be stable from −0.4 V to −0.75 V (compared to the normal hydrogen electrode as a reference; vs. NHE). Different shapes of the voltammogram were noticed when comparing with a solution saturated in N_2_, CO and CO_2_, associated with the suppression of the H_2_ flux due to a high negative potential. There was a decrease of current of ca. 10 and 20 mA.cm^−2^ from the N_2_-saturated to CO- and CO_2_-saturated solutions, respectively. On the other hand, there was no confirmation of the CO formation mechanism. Therefore, more conclusive experimental results are still necessary. Moreover, theoretical ab initio analyses have shown that monocrystalline mackinawite with specific {011} and {111} surface structures [136,137] strongly adsorbs CO_2_ molecules, while {001} is a more stable, yet less reactive structure [136]. Furthermore, the {111} structure has shown a major charge transfer, activating CO_2_ [136].

In addition, greigite, the product of mackinawite oxidation, is a metastable mixed-valence iron sulfide (${\mathrm{Fe}}^{\mathrm{II}}{\mathrm{Fe}}_{2}^{\mathrm{III}}S_{4}$) known to be widespread in freshwater [132,138]. The reduction of CO_2_ into C1-C3 products, such as methanol, plus formic and pyruvic acids, has been shown by experiments with carbon-supported greigite electrodes at room temperature and varied pH conditions, comparable to those of the early ocean models [97]. These products were detected under pH 4.5, 6.5 and 10.5, and also under low potentials—from 0.2 to −0.8 V compared to NHE—in an overall current (faradaic) efficiency from 0.54% to 8.11%. Greigite is considered thermodynamically stable below pH 7 in regions under potentials from 0.45 V to −0.2 V (vs. NHE) [132]. Experimental spectroelectrochemical investigations on synthetic greigite surface structures have also shown an interaction between this surface and CO_2_ under certain conditions that are necessary for its reduction [138]. In CO_2_-saturated solutions, under pH 4.5 and 10.5, greigite has shown lower efficiencies for CO_2_ reduction, due, respectively, to the passivation effects of the formation of FeCO_3_ on its surface and the iron oxide/hydroxide surface. Thus, under the pH = 6.5 condition, CO_2_ reduction into the mentioned organics has shown an optimum efficiency [139]. In addition, an ab initio analysis of monocrystalline greigite, with {011} and {111} surface structures, also elucidates the CO_2_ reduction electrocatalysis process and mechanism [97].

Pyrite is an iron disulfide, FeS_2_, well known for its stability and abundance on the surface of Earth [131]. In addition, natural pyrite as a working electrode on high CO_2_ pressure (50 atm) in a pH 7 phosphate-buffered electrolyte has shown the capacity for CO_2_ electro-reduction [140]. The linear sweep voltammogram has produced different shapes when comparing results under an Argon (Ar) atmosphere and in the presence of CO_2_ for a rotating pyrite electrode. The rotation of the electrode has an analogous effect to a flow-through system, eliminating reaction intermediaries from the surface due to hydrodynamic effects. When the potential was ca. −900 mV (vs. NHE), the presence of CO_2_ showed an increase of current of around 50 µA. Formate (${\mathrm{HCOO}}^{-}$) production on the pyrite surface showed an exponential increase with a potential of −0.8 V (vs. silver/saturated silver chloride electrode; Ag/AgClsat.d). However, faradaic efficiencies were up to 0.1% at −1 V. Nevertheless, under room pressure, pyrite has been shown to reduce CO_2_ into CO in potentials below −1 V (vs. NHE). The results related to the formation of organics on iron sulfides are demonstrated in Table 2. 

**Effects of Nickel on Iron sulfide minerals electrochemistry**. The presence of nickel (Ni) in the iron sulfide minerals has been shown to increase the activity of these minerals in the CO_2_ electro-reduction, compared to pure Ni or iron (Fe) sulfides [99,107]. Its higher efficiency for CO_2_ reduction electrocatalysis seems to be noteworthy for its incorporation into the AHV model. Ni-bearing mackinawite, in contrast to pure mackinawite, was envisaged as a CO_2_ reduction catalyst, as other Ni-containing iron sulfides are known to be [98].

The substitution of Ni for Fe in greigite results in the formation of the violarite (FeNi_2_S_4_) [141], an important mineral proposed to have been precipitated in the early vent–ocean interface [57]. Surprisingly, there are some electrochemical and voltametric results using violarite as a working electrode, characterizing some interface phenomena in acidic solutions in different pH conditions [139,142,143]. Analyzing Pourbaix diagrams, it is possible to see a wider stable region, comparable to the other minerals mentioned here, from 0.25 V to −0.6 V (vs. NHE). However, compared to greigite, different cycles in the voltammogram do not superpose. This results from the passivation effect due to the iron oxyhydroxide formation [139]. Hence, differently from greigite and mackinawite, there is a low proton and water reduction on the violarite surface from 0 to −1 V (vs. NHE).

In addition, violarite has been shown to adsorb CO_2_, increasing H_2_ generation and suppressing the passivation due to the formation of iron oxyhydroxide [139,141]. CO_2_ electroreduction on the violarite surface has been shown to initiate at ca. −500 mV [141], and its efficiency increases at 1.3 V (vs. NHE). Hence, at room temperature and pH 5.5, the efficiency has increased ~85-fold compared to greigite. The treatment of violarite with triethylamine and poly-allylamine hydrochloride has resulted in even higher efficiencies at the same potential (see Table 2).

Other iron [–nickel] sulfides, such as pyrrhotite (Fe_7_S_8_) and pentlandite ((Fe,Ni)_9_S_8_), have also shown activity as CO_2_ reduction catalysts under the early Earth conditions. Pyrrhotite is estimated to be the most abundant iron sulfide in the entire Solar system. Pyrrhotite and pentlandite have electrochemical behaviors comparable to those of greigite and violarite, respectively, when in a phosphate-buffered electrolyte [139]. Thus, both interact with aqueous CO_2_ for electroreduction that halts the formation of iron oxyhydroxide, though we still lack more details about these interactions.

**Other metal sulfides electrochemistry.** Besides not being mentioned directly in the AHV model, minerals such as pyrite, iron–copper and iron–zinc sulfides were explored for CO_2_ reduction electrocatalysis under hydrothermal conditions. Iron–copper and iron—zinc sulfides (Fe(Cu)S and Fe(Zn)S) were produced in the process of iron replacing in greigite using different metal/iron rates [141]. Although these minerals have shown capacity as CO_2_ reduction electrocatalysts under the early Earth physio-chemical conditions, their faradaic efficiency is much lower when compared to iron–nickel sulfides, such as violarite.

The surfaces of several other transition metal sulfides were investigated for CO_2_ reduction. Metal sulfides, such as Ag_2_S, CdS, CoS, CuS, FeS, MnS, MoS_2_, NiS, WS_2_, PbS and ZnS, have shown to perform CO_2_ reduction under induced potentials from −1.2 V to 0.8 V (vs. NHE) [144]. Under these conditions, transition metal sulfides have been shown to form CO and HCOOH in current efficiencies varying from, respectively, 0.1% to 42.5% and from 0.1% to 4.7%. Higher efficiencies were detected on CdS and CuS surfaces. Not only the proposal for the CO_2_ reduction in the AHV model is considered as a background for these works, but also the importance of a CO-rich environment for works in surface metabolism model is emphasized. In addition to CO_2_ reduction, MoS_2_ has also shown electrochemical activity for the reduction of nitrate (${\mathrm{NO}}_{3}^{-}$) and (${\mathrm{NO}}_{2}^{-}$) into ammonia (${\mathrm{NH}}_{3}$) [145]. 

#### 3.2.3. Microfluidic Scale Setup Simulating the Hydrothermal Vent-Ocean Interface 

As discussed in the last section, the formation of inorganic membranes associated with AHV mounds happens due to the juxtaposing of the two fluids involved in the process: the early ocean and the hydrothermal fluid. Recently, a microfluidic setup was used to study the chemical garden membrane in more controlled conditions. This Y-shaped lab-on-a-chip setup was presented as an alternative for understanding several properties of membrane formation through injection methods, such as hydrodynamic and diffusional effects, plus concentration distribution on the interface, also permitting in situ analysis [146]. Thus, instead of the injection method forming chemical gardens, the contact of fluids was used to simulate the interface between the early ocean and the hydrothermal fluid on a micro-scale. This setup was also an important alternative for the investigation—in a flow-through protocol—of the emergence of chemiosmosis [147]. Therefore, the same setup may be adapted to an experimental protocol to analyze products of the prebiotic chemistry in the hydrothermal–vent interface.

The formation of a microscale iron sulfide mineral precipitate was shown to be possible in a flow-through reactor under the hydrothermal interface conditions [148]. In this set-up simulating the AHV interface, a pH gradient was maintained even without the precipitate, due only to the laminar flow. In addition, in a more realistic model for the AHV interface, iron–nickel sulfide precipitate was used to reduce CO_2_ into HCOOH at room temperatures and moderate pressure (1.5 bar) [147]. In this model, several parameters were tested and a possible indirect mechanism for the reduction was evidenced, in which CO_2_ was reduced on the ocean side of the interface, while H_2_, on the vent side, was oxidized, probably transferring electrons through the barrier of iron [–nickel] sulfides.

## 4. Overall Perspectives and Trends for the Model

The main conceptual framework of the AHV model is the continuity of living system evolution as entropy converters. According to it, the emergence of a specific complex engine is considered a key factor for the nature of life and the understanding of its emergence. Therefore, the framework is based on complex systems and on far-from-equilibrium thermodynamics. Both have been shown to be extremely important in structuring the modern hypotheses for the EoL. In addition, this approach has been shown to be open for analytical and numerical models to supplement the conceptual models that have been established so far. This is incredibly important because it will, for sure, enlighten the nature of living systems compared to non-living ones. However, the inclusion of these models demands precision on the manipulation of concepts, and in several systems, they are knowingly difficult to be managed. This difficulty is clear even in the cases of classic systems that are considerably simpler than living systems are known to be. Accordingly, we must consider that living systems couple a myriad of phenomena that include both types of far-from-equilibrium dynamical behaviors: steady states and oscillations. Thus, describing living systems using tools of those subject areas, such as phase diagrams, finding trends in their topologies and finding results for key parameters, such as entropy production rate, is anything but trivial. However, it also does not seem to be an impossible task, as long as important constraints can be enforced. 

Hence, according to the most recent works, green rusts have emerged as an important class of minerals that are estimated, according to the AHV model, to have a central role as a seed for the engines that act in the maintenance of life, due to their peculiar structure. This can be an important direction for future experimental approaches, resulting in a notion around natural electrochemically induced mechanical phenomena, as occurs in the enzyme-mediated chemiosmotic mechanism, in the modeled early ocean–vent interface.

In addition, experimental approaches have resulted in important knowledge, obtained from constraining the possible components that are candidates for having participated in the emergence of the chemiosmosis in early AHVs. These experiments were mostly based on specific variants of hypotheses inspired in the AHV model, such as the early bioenergetics and the geoelectrochemical-driven origin of life. In the first group of experimental setups, chemical gardens were important to show the probable morphologies and composition of early AHVs. They also proved that a geoelectrochemical potential could have existed in those environments. In addition, they have shown that specific minerals of iron [nickel] sulfides, especially violarite, display more feasibility than iron–sulfur minerals in mediating the electrochemically induced CO_2_ reduction at the vent–ocean interface. However, an observation based on theoretical electrocatalysis has revealed that low current efficiencies can be increased by exploring the use of surface alteration with dopants and other effects. In summary, recent works have demonstrated important steps towards more realistic experimental simulations of the mentioned interface. In these simulations, the indirect mechanism for performing the first steps of the WL pathway at the vent–ocean interface—the H_2_ oxidation occurs on one side of the barrier of iron [–nickel] sulfide, while CO_2_ reduction occurs on the other side—has been demonstrated to be possible. Another relevant factor that seems to be an important step for further experiments, however, and that is still difficult to explore in a laboratory, is the very high pressure that is expected to have existed in the submarine scenario of early vents (above tens of MPa). Nonetheless, the mechanism can be better explored using experimental simulations by using bench-top reactors or a microfluidic setup.

Recent critiques have also given rise to important questions that may provide directions also to experimental works. As an example, in the works from Jackson (2016, 2017) [149,150,151], critical reviews for the AHV model were shown, displaying some important questions mainly regarding the geoelectrochemical-driven phenomena that are proposed in the model. Moreover, most recently, the so-called “water problem” has come to the fore [152,153,154]. This basically consists of the argument that in the conditions of the submarine scenarios, such as that of the AHV model, it would be difficult for life to emerge. This difficulty is argued to exist based on the results that show inhibition of the self-assembly of membranes caused by high salinity, which is proposed to occurs in early seawaters; and due the necessity of activated molecules to promote condensation for polymer synthesis in aqueous environments [153]. In the work of Russell (2021) [152], there is a more complete review on the critiques for the AHV model and it is presented responses for each one of them. A long and detailed discussion on these critiques would be outside the scope of our work. However, we want to bring attention to the fact that the work from Russell also shows a list of experiments/tests that can provide answers for the problems proposed. This list emphasizes how green rust minerals, such as fougerite, are a promising key for experiments inspired in the AHV model in future works.

Finally, the application of the main concept from the AHV model to investigate the EoL problem is also a trend to be explored. Considering the different conditions of the fluids that form the AHV mounds, models and experiments can be designed for that purpose. The geoelectrochemical-driven origin of life used the conceptual base of the emergence of chemiosmosis, which is applicable in the context of hot springs [155,156]. Nevertheless, it must not be forgotten that aqueous environments with radioactive minerals on the Hadean Earth have also been shown to be feasible for the adaptation of the AHV model [157].

## 5. Concluding Remarks

The discussion developed here has shown that the hypothesis involving the emergence of life in alkaline hydrothermal systems has benefited from a rich interdisciplinary approach for the open question on how life began on Earth. Since the discovery of the LCHF in 2001, the model has gained a lot of attention, and previous perspectives were widened. Concepts from geochemistry supplemented those from complex science and bioenergetics in a hypothesis that couples them based on the concept of continuity, as a consequence of the notion of emergence. The main conceptual framework of the AHV model, emphasizing the function and the nature of living systems, shows that it is still being revised. Accordingly, variants of the AHV model and hypotheses based on it have been presented as a very active frame of reference for experimental approaches that seek to test and constrain the hypothesis. The model described also provides a pathway to explore the possibility of the existence of living systems in other wet rocky bodies. In addition, the hypothesis has also been shown to feed models for the EoL in scenarios other than AHVs. Thus, several experimental protocols have been developed, and important results constrained the AHV hypotheses and can also help in fine-tuning them. This is an important aspect of the model, considering that it is still being constructed and proving to be actively enhanced and in a constant reviewing process.

## Figures and Tables

**Table 1:** Membrane potential for iron sulfide chemical gardens before and after the substitution of the membrane-forming injected solution for a Na_2_S solution inside of the precipitated membrane [118].

Basic Injection Method			
Reservoir Solution (Early Ocean Simulant) Composition	Injected Solution (Early Hydrothermal Fluid) Composition	Maximum Potential (mV)	
FeCl_2_ + FeCl_3_ + NaNO_3_	NaOH + CH_3_OH	1200	
FeCl_2_	Na_2_S	1400	
FeCl_2_ + NaNO_3_	NaOH	640	
Substitution of the interior fluid after the membrane formation			
Reservoir solution (early ocean simulant) composition	Injected solution (early hydrothermal fluid) composition	Maximum potential (mV)	Maximum potential after solution substitution ^a^ (mV)
FeCl_2_ + FeCl_3_	NaOH	431	881
	NaOH + K_4_P_2_O_7_	473	914
	NaOH + alanine	485	929
	NaOH + K_4_P_2_O_7_ + alanine	239	923

^a^ Substitution for a 50 mmol·L^−^^1^ Na_2_S containing solution.

**Table 2:** Iron minerals and the potentials of different products of CO_2_ reduction on their surface under several conditions.

Surface	Electrochemical Surface CO_2_ Reduction Product	Condition	Current Efficiency (%)	Reference
Fe_3_S_4_	CO	Room temperature; pH = 5.5; 760 torr CO_2_; 1.3 V (vs. NHE)	<0.01	Yamaguchi et al. (2014) [141]
	CH_4_		<0.01	
	Formic acid	Room temperature; room pressure; E from 0 to −1 V ^a^	0.1 (pH = 4.5) 1.51 (pH = 6.5) 22.43 (pH = 10.5)	Roldan et al. (2015) [97]
	Acetic acid		0.23 (pH = 4.5) 2.61 (pH = 6.5) 0.5 (pH = 10.5)	
	Methanol		0.21 (pH = 4.5) 1.21 (pH = 6.5)	
	Pyruvic acid		2.78 (pH = 6.5)	
Ni—Fe_3_S_4_	CO	Room temperature; pH = 5.5; pCO_2_ = 1 torr; E = 1.3 V ^b^	<0.015 ~0.15 (Fe/Ni = 1; w/TEA) ^c^ ~0.05 (Fe/Ni = 1; w/PAH) ^d^	Yamaguchi et al. (2014) [141]
	CH_4_		<0.01 (Fe/Ni = 5) ~0.075 (Fe/Ni = 1) ~0.25 (Fe/Ni = 1; w/TEA) ^c^ ~0.35 (Fe/Ni = 1; w/PAH) ^d^	
Pyrite	Acetic acid	Room temperature; pH = 7; pCO_2_ = 50 atm	~0.025 (E = 0.8 V) ^a^ 0.12 (E ~ 1 V) ^a^ ~0.09 (E = 1.2 V) ^a^	Vladimirov et al. (2004) [140]

^a^ potential vs. Ag/AgCl sat.d. ^b^ potential vs. NHE. ^c^ triethylamine. ^d^ poly-allylamine hydrochloride.

## Data Availability

Data sharing not applicable.
